# Vestibulo-Ocular Reflex Abnormalities in Posterior Semicircular Canal Benign Paroxysmal Positional Vertigo: A Pilot Study

**Published:** 2017-09

**Authors:** Tayyebe Fallahnezhad, Mansoureh Adel Ghahraman, Saeid Farahani, Reza Hoseinabadi, Shohreh Jalaie

**Affiliations:** 1 *Department of Audiology, School of Rehabilitation, Tehran University of Medical Sciences, Tehran, Iran.*; 2 *Department of Physiotherapy, School of Rehabilitation, Tehran University of Medical Sciences, Tehran, Iran.*

**Keywords:** Benign paroxysmal positional vertigo, Head impulse test, Semicircular canals, Vestibulo-ocular reflex

## Abstract

**Introduction:**

Benign paroxysmal positional vertigo (BPPV), involving the semicircular canals, is one of the most common diseases of the inner ear. The video head impulse test (vHIT) is a new test that examines the function of the canals. This study aimed to investigate the vestibulo-ocular reflex (VOR) gain, gain asymmetry and saccades after stimulating all six canals in patients definitively diagnosed with posterior semicircular canal BPPV (PSC-BPPV).

**Materials and Methods:**

Twenty-nine unilateral PSC-BPPV patients with normal oculographic and caloric results were enrolled in this study. vHIT was performed on six canals, and VOR gain, gain asymmetry and saccades were measured.

**Results:**

Sixteen (55.17%) patients had abnormal posterior canal VOR gains in the ipsilesional ear. VOR gains in both horizontal canals were within normal limits. Superior canal VOR gains were mostly lower than normal and were not correlated to PSC abnormalities (P>0.05). No corrective saccades could be observed.

**Conclusion:**

VOR gain in the direction of the posterior semicircular canal may be reduced in PSC-BPPV patients. Evaluation of PSC-VOR parameters could be beneficial, although superior canal measurements should be interpreted with caution.

## Introduction

Benign paroxysmal positional vertigo (BPPV) is one of the most common diseases of the inner ear. With a prevalence of 10.7–64 cases per 100,000 population, it is considered the most common type of vertigo. The female-to-male ratio of BPPV patients has been reported as 2:1 to 3:1. BPPV is characterized by a sensation of a short-time spinning (within less than 1 minute) which occurs due to the position of the head in relation to gravity. There may be signs such as nausea and vomiting, but it is not accompanied by hearing loss, tinnitus or neurologic symptoms (1). In 60–90% of cases, BPPV involves the posterior semicircular canal (2), but also occurs in the anterior or horizontal semicircular canals. Diagnosis of BPPV is simply made using the Dix-Hallpike maneuver or a roll test (3). Since BPPV has different etiologies that may involve other parts of the vestibular system and may occur with another disease, it is necessary to evaluate peripheral vestibular function in suspected cases. Previous studies have shown caloric hypoactivity (4), tilting toward the affected side in a subjective visual vertical test (5), abnormal ocular and cervical vestibular-evoked myogenic potentials (6–9), and abnormal postural stability (10) in BPPV patients.

The video head impulse test (vHIT) is a new, although widely used, test that examines the function of the canals through observation and recording of eye responses during head movement in both the horizontal and vertical axes. This test was first introduced by Halmagy and Curthoys in 1988 for evaluating vestibulo-ocular reflex (VOR), and at first it was used for identifying damage to the lateral semicircular canal (11). The vHIT, caloric and rotary chair tests examine different frequency ranges (0.003 Hz in the caloric test; 0.1–0.64 Hz in the rotary test and 3–5 Hz in the vHIT). Horizontal vHIT has been reported to be normal in BPPV patients (12,13). In vertical vHIT, VOR gain asymmetry was found in patients with superior semicircular canal involvement (4). Some abnormal results were reported in patients with posterior semicircular canal BPPV (PSC-BPPV) (14).

It is important to investigate and report VOR changes using the horizontal and vertical results of vHIT in patients with posterior semicircular canal BPPV. Therefore, this study aimed to investigate the VOR gain, gain asymmetry and saccades after stimulating all six canals in patients affected by posterior semicircular canal BPPV.

## Materials and Methods

Twenty-nine patients (18 women and 11 men) diagnosed with unilateral posterior semicircular canal BPPV (PSC-BPPV) were included in this study. All patients gave written informed consent. The study was approved by the Human Research Ethics Committee of Tehran University of Medical Sciences.

Included patients had no other vestibular disorder based on a medical history and videonystagmography (Eye Dynamics, USA). In all patients, PSC-BPPV was confirmed through clinical symptoms and positive Dix-Hallpike maneuver. Patients were seated on the examination table, with their head turned (45 degrees) toward the ear to be tested and lowered to the table such that the head hung 20 degrees from the table. Observation of up-beating and torsional nystagmus toward the testing ear with true vertigo was an indication of PSC-BPPV. A roll test was also performed to exclude horizontal semicircular canal involvement. The patient lay on the table with the head flexed at 30 degrees and the neck supported by the examiner. The patient’s head was quickly rolled to one side and then to the other. If there were any horizontal nystagmus or the patient reported any vertigo, he/she was excluded from the study.

For VOR evaluation, a video system (vHIT GN Otometrics, Denmark) was used. The patient wore a pair of lightweight goggles which were tightly fitted onto his/her head. The goggles were equipped with a high-speed video camera (250 Hz), and the image of the right eye was reflected from a mirror to the camera. The eye was illuminated by two infrared light-emitting diodes. A sensor on the goggles measured head movement. Before initiation of the test, the eye position was calibrated. The patient was asked to fixate at an earth-fixed target, 90–100 cm in front of him/her. The experimenter gave the patient approximately 20 unpredictable, brief and abrupt head impulses (10–20 degrees), unpredictably turning to the left or right on each trial. To examine the vertical canals, the patient’s head was turned 35–45 degrees to the right for testing in the left anterior/right posterior (LARP) plane or 35–45 degrees to the left for testing in the right anterior/left posterior (RALP) plane while standing at the same fixed target, and the head impulses were delivered at the pitch axis. After completion of testing each semicircular canal, the outcome parameters (head-velocity stimuli and eye velocity responses) were displayed on the computer screen, and a graph of the calculated VOR gain (ratio of eye velocity to head velocity) for every head impulse was shown. A VOR deficit was defined as a vHIT gain of <0.8 for lateral canals and <0.7 for vertical canals (15). In all cases, gain asymmetry was calculated using the same formula: (GSCc–GSCi)/ (GSCc+GSCi). A positive value indicated that the contralesional gain was greater than the ipsilesional, and *vice versa*.

All tests were performed on the same day with an interval of 1–2 hours between the Dix-Hallpike maneuver and vHIT.

## Results

We assessed 29 patients (18 female, 11 male; ratio: 1.63), with a mean age of 50.86 years (range: 16 to 77 years). All patients had unilateral PSC-BPPV. The left ear was affected in 12 patients (41.4%) and the right ear in 17 patients (58.6%). No spontaneous nystagmus, abnormal oculomotor function or canal paresis was detected. VOR evaluation in the direction of the affected PSC showed that there were 16 patients (55.17%) with abnormal gain (<0.7) and 13 patients (44.82%) with normal gain (≥0.7). No patients had abnormal VOR gain in the contralesional posterior canal. Sixteen patients had abnormal anterior canal VOR gain in the ipsilesional side and 24 showed abnormal results in the contralesional side. All patients demonstrated normal VOR gain in the evaluation of horizontal semicircular canals on both sides. 

**Fig1:**
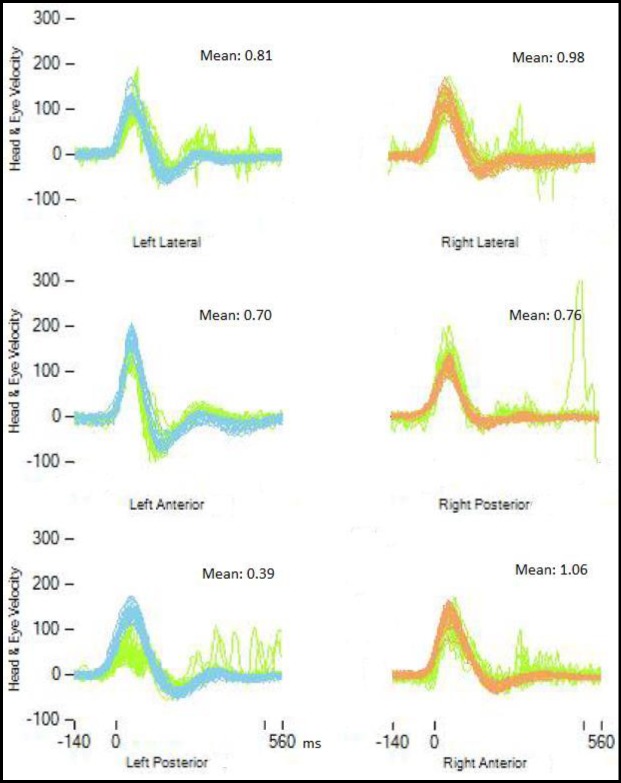
Representation of video head impulse test (vHIT) results in the left posterior semicircular canal benign paroxysmal positional vertigo. All gain values are presented in the box over the right corner of each test. vHIT was performed in the direction of all semicircular canals. Light green lines represent the eye movement velocity

Fig. 1 represents the vHIT graphs obtained from a patient with abnormal PSC-VOR gain. No corrective saccades could be observed in any of the patients in the examination of all semicircular canals. VOR gain for all semicircular canals is shown in Table 1. There were significant differences between PSC-VOR gains on the two sides (P=0.024) (Fig. 1).

**Table 1:** Mean (standard deviation) VOR gain in the direction of all semicircular canals in patients with and without abnormal posterior canal VOR gain

**Semicircular canal**	**VOR gain (SD)**		
	**Ipsilesional semicircular canal**	**Contralesional semicircular canal**	**p**
Posterior	0.66 (0.14)	0.77 (0.07)	0.024
Anterior	0.61 (0.14)	0.54 (0.15)	0.167
Horizontal	0.91 (0.08)	0.90 (0.08)	0.689

VOR: vestibulo-ocular reflex

As indicated by the Pearson correlation test, the VOR gains in the anterior canal were not significantly correlated to the PSC-VOR gains abnormality in either the affected or unaffected sides; Pearson’s correlation coefficient was 0.07 (P=0.71) and 0.26 (P=0.15), respectively. Although recurrence of BPPV appeared more prevalent in patients with abnormal PSC-VOR gain than in patients with normal gain, the difference was not significant (69.23% vs 30.76%, P=0.170).

Table 2 reports gain asymmetry between the pairs of canals on both sides. The widest range of asymmetry was in ipsilesional PSC-VOR gains compared with contralesional anterior canal VOR gains. The narrowest range was between the horizontal canals on both sides.

**Table 2:** Mean gain asymmetry between ipsi-and contralesional semicircular canal VOR gains.

Semicircular canals	Mean (SD)	Range (min–max)
HSCi-HSCc	0.009 (0.41)	0.18 (−0.07–0.10)
PSCi-PSCc	0.048 (0.09)	0.40 (−0.21–0.19)
ASCi-ASCc	0.075 (0.16)	0.58 (−0.03–021)
PSCi-ASCc	0.016 (0.11)	0.67 (−0.52–0.15)
PSCc-ASCi	0.131 (0.17)	0.44 (−0.06–0.38)

HSC: Horizontal semicircular canal; PSC: Posterior semicircular canal; ASC: Anterior semicircular canal; i: ipsilesional, c: contralesional

## Discussion

Video head impulse is a new test that allows quantitative assessment of semicircular canal function and provides specific information about angular VOR. We evaluated the VOR responses in BPPV patients with posterior canal involvement. Patients included in this study showed normal oculomotor and caloric responses. Twenty-nine patients with PSC-BPPV (mean age=50.86 years) were assessed. The right ear was involved to a greater extent, which is similar to other reports and may be related to the patient sleep position (16,17). BPPV was more frequent in females, as was also reported in previous studies (18,19).

In our results, PSC-VOR gain in the direction of the affected ears was abnormal in 16 patients. Free floating particles in the posterior semicircular canal that disturb endolymphatic flow lead to VOR and gaze stabilization deficiency. Previous studies have shown higher VOR gain asymmetries in vertical canal BPPV (14,20). Abnormal PSC function resulting from higher maximum slow phase velocity of head shaking nystagmus and reduced time constants have been reported previously in 19% of patients with vertical canal BPPV (21). Abnormal PSC-VOR gain in patients with isolated loss of the right posterior semicircular canal was also reported (15).

 All patients in this study showed normal VOR gain in the horizontal canal. Since the dislodged otoconia were floating in the PSC, normal function in the horizontal canal was expected. All patients had normal caloric responses. There is the possibility of abnormal horizontal semicircular canal VOR gain in BPPV patients with hypoactive caloric responses. In other studies, normal HSC-VOR gain in patients with superior or posterior semicircular canal BPPV was attributed to high incidence of spontaneous recovery in this type of BPPV; therefore, spontaneous recovery requires normal VOR gain in HSC (4,20).

Superior semicircular canal (SSC) VOR gain on both sides exhibits normal and abnormal values that are not significantly correlated to posterior semicircular canal VOR gain (P>0.05). Given that abnormal VOR gain in SSC was also seen in our normal cases (data not shown), it seems that the manner of eye movement recording lead to illusive results in SSC gain; therefore, VOR gain in SCC should be interpreted with caution. McGarvie et al. and Halmayi et al. reported that eyelid interference with the pupil image is a major artifact that disturbs the vertical canal response. Head movement in the direction of the vertical canal (especially the anterior semicircular canal) causes the eyelid to obscure part of the pupil. This changes the eye velocity response (22,23). Moreover, in the current study, a large number of abnormal values in SSC-VOR gain were seen on the contralesional side, and in 60% of cases, the contralesional side was the left ear. Different vertical translation was seen between the right and left eye during testing of the SSC that may be due to the test protocol, in which the camera recorded only right-eye movement. The increased vertical translation of the right eye lead to greater eye rotation to provide gaze stability and finally increased VOR gain in the right anterior canal (24). The examiner’s experience is that another factor that may influence the results.

In our study, no corrective saccade was recorded in any patients. There is a probability that a severe amount of vestibular damage causes the occurrence of corrective saccade (12). This type of result (normal/abnormal gain without saccade) has been reported previously (12). Corrective saccade in BPPV could be related to coincidence of a baseline problem along with BPPV (13).

Recurrent BPPV was more frequent (70%) in patients with abnormal VOR gain rather than in those with normal VOR gain (30%), although the difference was not significant. Reduced saccular function in BPPV was reported in other studies (8,25,26). It has been demonstrated that abnormality in the cervical vestibular-evoked myogenic potential (cVEMP) and ocular VEMP (oVEMP) was more prevalent in patients with recurrent BPPV, and also VEMP abnormality is a risk factor for recurrence of BPPV (7). Identical polarity of hair cells in PSC crista and the inferior half of the saccular macula allows a relationship between the otolith and canal in the medial vestibular nucleus. The inhibitory effect of saccule on the vestibulo-ocular reflex originated from semicircular canals, leading to VOR deficit. Saccule dysfunction is severe in recurrent BPPV (27); therefore, VOR deficiencies may be more prevalent in recurrent BPPV. It should be noted that we did not evaluate otolith function in our patients. In addition, our findings may be due to the small number of patients in the two groups.

## Conclusion

VOR gain in the direction of the posterior semicircular canal can be reduced in PSC-BPPV patients. Testing SSC in vHIT is beneficial but it tends to be more affected by artifacts; therefore, it must be done with great precision.
